# The Saposin-Like Protein AplD Displays Pore-Forming Activity and Participates in Defense Against Bacterial Infection During a Multicellular Stage of *Dictyostelium discoideum*

**DOI:** 10.3389/fcimb.2018.00073

**Published:** 2018-03-15

**Authors:** Ranjani Dhakshinamoorthy, Moritz Bitzhenner, Pierre Cosson, Thierry Soldati, Matthias Leippe

**Affiliations:** ^1^Zoological Institute, Comparative Immunobiology, University of Kiel, Kiel, Germany; ^2^Department of Cell Physiology and Metabolism, Faculty of Medicine, University of Geneva, Geneva, Switzerland; ^3^Department of Biochemistry, Faculty of Science, University of Geneva, Geneva, Switzerland

**Keywords:** amoebapore, antimicrobial peptides, *Dictyostelium discoideum*, host-pathogen interactions, saposin-like proteins, slugs, Sentinel cells, innate immunity

## Abstract

Due to their archaic life style and microbivor behavior, amoebae may represent a source of antimicrobial peptides and proteins. The amoebic protozoon *Dictyostelium discoideum* has been a model organism in cell biology for decades and has recently also been used for research on host-pathogen interactions and the evolution of innate immunity. In the genome of *D. discoideum*, genes can be identified that potentially allow the synthesis of a variety of antimicrobial proteins. However, at the protein level only very few antimicrobial proteins have been characterized that may interact directly with bacteria and help in fighting infection of *D. discoideum* with potential pathogens. Here, we focus on a large group of gene products that structurally belong to the saposin-like protein (SAPLIP) family and which members we named provisionally Apls (amoebapore-like peptides) according to their similarity to a comprehensively studied antimicrobial and cytotoxic pore-forming protein of the protozoan parasite *Entamoeba histolytica*. We focused on AplD because it is the only Apl gene that is reported to be primarily transcribed further during the multicellular stages such as the mobile slug stage. Upon knock-out (KO) of the gene, *aplD*^−^ slugs became highly vulnerable to virulent *Klebsiella pneumoniae*. *AplD*^−^ slugs harbored bacterial clumps in their interior and were unable to slough off the pathogen in their slime sheath. Re-expression of AplD in *aplD*^−^ slugs rescued the susceptibility toward *K. pneumoniae*. The purified recombinant protein rAplD formed pores in liposomes and was also capable of permeabilizing the membrane of live *Bacillus megaterium*. We propose that the multifarious Apl family of *D. discoideum* comprises antimicrobial effector polypeptides that are instrumental to interact with bacteria and their phospholipid membranes. The variety of its members would allow a complementary and synergistic action against a variety of microbes, which the amoeba encounters in its environment.

## Introduction

Amoebozoa are interesting models to study the early evolution of innate immunity (Leippe, 1999). The social amoeba *Dictyostelium discoideum*, a genetically tractable model for the study of cell biology, has recently become a powerful model organism in infection biology. In particular, it has been employed as a surrogate host for human pathogens such as *Legionella* (Farbrother et al., 2006), Mycobacteria (Hagedorn and Soldati, 2007; Hagedorn et al., 2009), and *Pseudomonas* (Cosson et al., 2002; Alibaud et al., 2008). In its amoebic stage, *D. discoideum* phagocytoses microbes and thereby resembles phagocytes of the innate immune system (Cosson and Soldati, 2008). Upon starvation, the amoebae aggregate and undergo a programmed differentiation and morphogenesis. One of the specific stages is the so-called slug, formed by the aggregation of about 100,000 amoebae, which will eventually differentiate into a fruiting body. Among slug cells, Sentinel cells represent a simple and efficient immune system. These phagocytes patrol the slug body to capture toxic compounds and invading bacteria. The Sentinel cells are continuously shed behind in the slime sheath of migrating slugs (Chen et al., 2007). These immune-like phagocytes have recently been reported to possess the capacity to produce extracellular DNA traps around the pathogen/foreign body (Zhang et al., 2016) in a way similar to phagocytes of vertebrates and invertebrates (Brinkmann et al., 2004; Robb et al., 2014). These features make of *D. discoideum* an even more attractive model to trace back the conserved functions of the innate immune system across evolution from protozoans to metazoans (Chen et al., 2007; Hagedorn et al., 2009; Zhang and Soldati, 2016).

Despite these promising results, the knowledge about the arsenal that *D. discoideum* employs to kill phagocytosed microbes and to combat potential pathogens is still scarce. The only example of an antimicrobial effector characterized at the protein level is the AlyA lysozyme, which had been isolated from amoebic extracts and found to be able to degrade bacterial cell walls (Müller et al., 2005).

According to the information derived from the genome project, *D. discoideum* possesses at least 15 genes potentially coding for lysozymes belonging to several different classes of these hydrolytic enzymes (Eichinger and Noegel, 2005; Müller et al., 2005). Beside the family of Alys, putative *Entamoeba*-type lysozymes, phage-type lysozymes, and C-type lysozymes (LyCs) can be identified in databases (Müller et al., 2005).

Another multifarious gene family of *D. discoideum*, which products may target bacterial membranes and thereby kill bacteria directly, is the one coding for saposin-like proteins (SAPLIPs). Structurally, SAPLIPs are characterized by four or five compactly packed alpha-helices, and are typically stabilized by three disulfide bonds built by a conserved array of six cysteine residues (Liepinsh et al., 1997). Functionally, SAPLIPs fulfill various biological functions, but the members of this family have in common that they interact with lipids and membranes (Munford et al., 1995; Bruhn, 2005; Kolter et al., 2005). SAPLIPS with antimicrobial activity can be found in phylogenetically diverse organisms ranging from protozoans to mammals (Leippe et al., 1991; Andersson et al., 1995; Peña et al., 1997).

In pathogenic amoebae, saposin-like proteins are well-known as pore-forming proteins that permeabilize the membranes of bacteria and human host cells (Leippe, 2014). The most comprehensively studied member of amoebic SAPLIPs is amoebapore A from *E. histolytica*, the tertiary structure revealed the characteristic SAPLIP fold (Hecht et al., 2004). We therefore provisionally termed the putative SAPLIPs of which *D. discoideum* Apls for amoebapore-like peptides.

*Dictyostelium discoideum* possesses 17 Apl genes that potentially can give rise to 33 SAPLIP peptides given that larger precursor proteins containing more than one SAPLIP domain (also termed saposin B domain) might be processed to release several mature SAPLIPs, as exemplified for the name-giving saposins (O'Brien and Kishimoto, 1991) and for naegleriapores of the free-living amoeba *Naegleria fowleri* (Herbst et al., 2004). Such an enormous variety of SAPLIPs in one species is only known so far from *C. elegans*, a nematode that also feeds on microbes (Roeder et al., 2010).

*In D. discoideum*, one may speculate that these proteins act complementarily and synergistically and constitute an important part of the antimicrobial armamentarium during its unicellular and multicellular stages. Nonetheless, functions other than killing of bacteria by membrane permeabilization have been reported for amoebic SAPLIPs (Michalek and Leippe, 2015).

In the present study, we have chosen AplD among the plethora of potential *D. discoideum* SAPLIPs for a more detailed functional study because it became apparent that: (i) the primary translation product comprises a single SAPLIP domain preceded by a putative signal peptide as known for amoebapores; (ii) the gene has been reported to be differentially expressed upon bacterial challenge; and (iii) most importantly with respect to immunity, *aplD* is reported to be primarily transcribed during the multicellular stages (Dicty express: https://dictyexpress.research.bcm.edu/bcm/#/all?genes=DDB_G0293010).

At the protein level, we characterized the ability of AplD to permeabilize the membranes of live bacteria and liposomes by monitoring quantitatively the activity of a recombinantly expressed protein (rAplD). *In vivo*, we phenotypically analyzed the effect of ablation of *aplD* on amoebic growth on various bacterial lawns, on bacterial killing in the amoebic stage, and on the slug's capacity to fight a bacterial infection.

## Materials and methods

### Bacterial cultures

Various bacterial strains were used in the study (Supplementary Table 1). The bacterial cultures were grown at 220 rpm at 37 °C for 12 to 14 h in Luria-Bertani medium.

### *Dictyostelium discoideum* cultures

Ax2 obtained from the Dicty stock center was used for generating the *apl* and *lyC* KO mutants. Ax2 amoebae were grown in maltose-HL5 medium and blasticidin (8 μg/ml)-containing maltose-HL5 medium was used to select the KOs. The *D. discoideum* cells transfected with prestalk reporter (*ecmAO*-RFP), prespore reporter (*pSA*-RFP) plasmids, and *aplD*^−^/[act6]:*aplD*.FLAG cells [rescue strain; mentioned as *aplD*^−^(+)] were cultured in maltose-HL5 medium complemented with G418 (10 μg/ml) and blasticidin (8 μg/ml).

### Creation of KO vectors

Short gene fragments from the 5′ and 3′ regions of *apl* and *lyC* genes were amplified by PCR and ligated appropriately at the 5′ and 3′ flanking ends of the blasticidin resistance (Bsr) gene present in the pLPBLP gene disruption vector as described in Faix et al. (2004). Before transfection, the KO vectors were subjected to DNA sequencing (Eurofins MWG operon, Germany) to verify vector orientations and mutations.

### Plasmids

pLPBLP, *ecmAO*-RFP, *pSA*-RFP, and pD*neo*2a-3xFLAG plasmids were received from the Dicty stock center.

### Targeted gene ablations in *D. discoideum*

Gene disruptions were achieved by homologous recombination between the respective *apl*s and *lyC*s with their corresponding KO vectors. Ax2 cells (2 × 10^7^) were sedimented at 560 × *g* at RT for 5 min. The pellet was resuspended in 1 ml ice-cold electroporation (EP) buffer (10 mM K_2_HPO_4_, 10 mM KH_2_PO_4_, 50 mM sucrose, pH 6.2.), washed at 10,000 × *g* at RT for 2 min, and briefly incubated on ice. The respective linearized KO vector was resolved in 100 μl ice-cold EP^++^ buffer (EP buffer containing 1 mM MgSO_4_, 1 mM NaHCO_3_, 1 μM CaCl_2_, and 1 mM ATP, pH 6.2) and subsequently mixed with Ax2. Immediately, this cell mixture was transferred to 2-mm gap electroporation cuvette (BTX electroporation cuvettes, Harvard apparatus) and electroporation was carried out at 300 V, 2 ms time constant with five square pulses, including 5-s intervals inbetween the pulses, using a BTX 830 electroporator (Harvard apparatus). After 10 min incubation on ice, the cell suspension was transferred to Petri dishes that contain 10 ml maltose-HL5 medium and incubated at 22°C. Blasticidin (8 μg/ml) selection was introduced 24 h later. Blasticidin-resistant clones had appeared after a week and were aspirated from the Petri dishes and seeded in 24-well plates (both from Sarstedt, Germany) for PCR analyses.

### Identification of KO clones

Cell lysates were prepared from the blasticidin-resistant clones following the method described in Charette and Cosson (2004). As described in Faix et al. (2004) three different PCR analyses were performed to verify the *apl* and *lyC* KO vectors insertion in 5′–3′ orientation at the *apl* and *lyC* loci. PCR screening results for *aplD* KO clones are shown in the Supplementary Figure 1. KO vectors (vector control, VC) and Ax2 genomic DNA (wild-type, WT) were used as respective controls for PCR analyses. The KO clones were confirmed for gene ablation at the respective gene locus by performing Southern hybridization (Supplementary Figure 2), using the DIG-High prime DNA labeling and detection starter kit II (Roche), following manufacturer's instructions. A probe was generated for the Bsr-resistance gene sequence, which spans for 249 base pairs and dioxygenin (DIG) was used as the labeling agent.

### Construction of an *aplD* rescue strain

The cDNA sequence of *aplD* was amplified by PCR and cloned under the Actin 6 promoter (*act6*) of pDneo2a-3xFLAG vector (Dubin and Nellen, 2010). The 3′ end of *aplD* cDNA was fused to the FLAG gene. Finally, the pDneo2a-3xFLAG[*act6*/*aplD*] plasmid was transfected into *aplD*^−^ cells. AplD-FLAG fusion protein expression in the rescue strain [*aplD*^−^(+)] was confirmed by Western blot analysis (Supplementary Figure 3). After separation by SDS-PAGE, proteins were transferred onto a polyvynilidene fluoride membrane and the blot was incubated with a mouse anti-FLAG M2 monoclonal antibody (Sigma Life Science) and subsequently developed using a goat anti-mouse IgG (H+L) conjugated to alkaline phosphatase (Jackson ImmunoResearch).

### *AplD* transcriptional profiling

To analyse transcription in axenic cultures, RNA was isolated from axenically grown *D. discoideum* amoebae (5 × 10^6^ cells) and the first strand cDNA was synthesized using the RNA template (cDNA synthesis kit, Invitrogen). Quantitative RT-PCR (qRT-PCR) was performed using the cDNA template as described by the manufacturer (qRT-PCR mix, TAKARA and Light cycler, Roche). For analysis of xenic cultures, Ax2 cells were mixed with bacteria (ratio 1:10) and the amoebae-bacteria cell suspensions were incubated at 140 rpm and 22°C for 8 h. Subsequently, the xenic Ax2 cells were washed three times at 425 × *g* at 4°C for 2 min to remove excess bacteria. cDNA was synthesized and qRT-PCR analyses were performed as described above. *AplD* transcription in axenic Ax2 amoebae was considered as reference to determine the *aplD* expression in xenic Ax2. Each symbol denotes individual experiment. The house keeping genes tested were *Ig7* (*rnlA*) and Glyceraldehyde-3-phosphate dehydrogenase (*GAPDH*). The bacterial strains tested include *Kp*LM21, *B. subtilis*, and PT531 (see Supplementary Table 1). For analysis during development, axenic Ax2 cells were washed with Sörensen's buffer (SB) at 510 × *g* and 22°C for 7 min. The cell pellets were resuspended in 100 μl SB and deposited on SB agar (1%) plates. Subsequently, the plates were incubated in a dark, moist chamber at 22°C and RNA was isolated from the starving amoebae (0 h), streaming cells (8 h), mounds (12 h), slugs (16 h), and fruiting bodies (24 h) and qRT-PCR was performed in duplicates. *AplD* expression in the starving amoebae (0 h) was considered as reference to quantify the *aplD* expression at other stages of development (Sillo et al., 2008). Three independent development experiments were performed and the bars represent standard deviation.

### Growth of *Dictyostelium* on bacterial lawns

Bacterial growth assays were performed on routinely used SM agar medium, which was devoid of glucose. SM agar plates were prepared as described in Froquet et al. (2009). Varying number (10^4^, 10^3^, 10^2^, and 10^1^) of *D. discoideum* amoebae were deposited on the bacterial lawn layered on SM agar plates and incubated in the dark at 22°C. Five days later, the KOs were scored for their plaque forming abilities by having Ax2 as comparative controls. Three independent experiments were performed with triplicates (see Supplementary Table 1 for details on the bacterial strains tested).

### Intracellular killing of bacteria by *Dictyostelium*

*Dictyostelium discoideum* amoebae were mixed with bacteria (100:1) and incubated at 140 rpm, 22°C. The total number of viable bacteria was measured at the indicated time points by following the method detailed in Benghezal et al. (2006). The rate of killing of *K. pneumoniae* bacteria by amoebae was quantified by counting the number of colony forming units (CFU) at each time point. Three independent experiments were performed.

### *Dictyostelium discoideum* development

*Dictyostelium discoideum* cells were washed with SB and deposited on SB agar (1%) plates at a cell density 5 × 10^5^ cells/cm^2^. Subsequently, plates were incubated in a dark, moist chamber at 22°C. The morphogenetic stages were imaged under the stereomicroscope (Olympus ULWCD 0.30) at time points mentioned (Supplementary Figure 4). *Dictyostelium discoideum* development on SB agar was monitored in three independent experiments.

### Prestalk (pst) and prespore (psp) slug patterns

Slug pattern analyses were performed as described in Parkinson et al. (2009). For prestalk reporter (*ecmAO*-RFP) examination, *D. discoideum* amoebae carrying the *ecmAO*-RFP plasmid were mixed with *D. discoideum* amoebae at 20:80 ratio and seeded on a SB agar (1%) plate at a cell density 5 × 10^5^ cells/cm^2^. For prespore reporter (*pSA*-RFP) examination, *D. discoideum* amoebae (20%) were mixed with *D. discoideum* amoebae marked with *pSA*-RFP (80%) and deposited on SB agar plates. The plates were incubated in a dark, moist chamber at 22°C until the migrating slugs were formed and were imaged under the stereomicroscope (Olympus ULWCD 0.30) (Supplementary Figure 4). Two independent experiments were performed in duplicates and at least ten slugs were imaged per plate.

### Slugs infection

*Dictyostelium discoideum* cells were harvested from Petri dishes, 2.5 × 10^7^ cells were sedimented, and the pellets were resuspended in 50 μl SB. This high density cell suspension was deposited on SB agar (1%) plates and incubated in a dark, moist chamber with an unidirectional light source until migrating slugs were formed (~18 h). Slug infection experiments were performed following Chen et al. (2007) with some modifications. The slugs were injured with a sterile needle (23G × 1^1/4^, B|BRAUN Injekt F) and a dense bacterial suspension, which was prepared from an overnight culture, was layered on the injured slugs. *K. pneumoniae* expressing GFP reporter (*Kp*GFP) and *E. coli* expressing DsRed reporter were used for infecting the injured slugs (see Supplementary Table 1). The slugs infected with *Ec* DsRed were imaged under the stereo microscope (Olympus ULWCD 0.30) 8 h post infection and those slugs infected with *Kp*GFP were imaged 20 h and 24 h post infection. *Kp*GFP was grown with ampicillin (1 mg/ml) for 12 h at 37°C. *Ec* DsRed was grown with isopropyl β-D-1-thiogalactopyranoside (IPTG, 100 mM) and kanamycin (150 μg/ml). Three independent experiments were performed and at least ten slugs were imaged per experiment for each strain [Ax2, *aplD*^−^, and *aplD*^−^(+)].

### Sentinel cells examination

*Dictyostelium discoideum* cells were allowed to form slugs on SB agar (1%) plates containing ethidium bromide (EtBr, 3 μg/ml) as described in Chen et al. (2007). After 3 h, Sentinel cells present in the migrating slugs were visualized under the stereomicroscope (Olympus ULWCD 0.30). Three independent experiments were performed and at least ten slugs were imaged per experiment for each strain (Ax2 and *aplD*^−^).

### Recombinant protein production and purification

The nucleotide sequence encoding putatively mature AplD (DDB0216216) was codon optimized for bacterial expression and synthesized by GeneArt (Regensburg, Germany). The cDNA was ligated into the pET-32a (+) (Novagen) expression vector containing an ampicillin resistance gene using *KpnI* and *XhoI* cleavage sites. The resulting plasmid encodes a fusion protein, which comprised an N-terminal thioredoxin followed by a hexahistidine (His_6_) tag and contained thrombin and tobacco etch virus (TEV) protease cleavage sites preceding the primary structure of AplD. TEV cleavage yielded a product with the amino acid sequence as follows: GEIDNNQCQICELLVKDIIEGLTANQSVEVIEHGLNLICDHIPLHVRVCKQFVDSNFQKIVQFIENHDDPQEICEKCGVC. The protein was recombinantly expressed in *E. coli* C 43 at 37°C after induction with 0.5 mM IPTG for 6 h and extracted by sonication on ice (Sonoplus HD 2200 sonicator, MS-73 titanium microtip, Bandelin electronic GmbH, Germany). The fusion protein in the soluble fraction was purified by immobilized-metal affinity chromatography (IMAC) using a TALON resin (Clontech, Saint-Germain-en-Laye, France) and subsequent anion-exchange chromatography (1-ml Resource Q column, GE Healthcare) using an Äkta purifier system (model P-900; GE Healthcare) and 20 mM Tris-HCl, pH 7.0 with a continous linear gradient of 0-1 M NaCl for elution. The N-terminal tag was removed by cleavage with TEV protease (ProTEV Plus protease, Promega) at 30°C overnight. The cleaved product was subjected again to IMAC and anion-exchange chromatography (Mini Q column 4.6/50, GE Healthcare, Solingen, Germany) to remove the fusion partner and the TEV from recombinant AplD. After final purification, the apparently homogeneous protein fraction was lyophilized, redissolved, and dialyzed against 10 mM sodium phosphate, pH 6.8.

Purity was proven by tricine-SDS polyacrylamid gel (SDS PAGE) using 13% separation gels (Schägger and von Jagow, 1987) and SeeBluePlus2 (Invitrogen) as standard protein marker for molecular masses. Protein concentration was determined with the BCA assay (Pierce, Thermo Scientific, Bonn, Germany). The molecular mass of recombinant AplD was determined by mass spectrometry in linear mode using a 4700 Proteomic Analyzer MALDI-TOF/TOF mass spectrometer (Life Technologies, Darmstadt, Germany).

### Assays for membrane-permeabilizing activities

Pore-forming activity using the liposome depolarisation assay (Leippe et al., 1991) and permeabilization of cytoplasmic membranes of live bacteria using the fluorescent dye SYTOX Green (Herbst et al., 2002) were measured as described previously. Briefly, in the liposome-depolarization assay a valinomycin-induced diffusion potential across the membrane of liposomes prepared from asolectin, a crude mixture of soy bean phospholipids, resulted in quenching of the enclosed fluorescent dye. Application of a pore-forming protein disrupts the membrane potential and the fluorescence intensity increases by the release of the dye. An increase of 5% within 1 min after addition of the pore-forming agent at 25°C is defined as one activity unit. In the membrane-permeabilization assay the fluorescent dye SYTOX Green (Invitrogen) is added to *Bacillus megaterium, E. coli, Klebsiella aerogenes, K. pneumonia Kp*LM21, or *K. pneumoniae K*^−^ as viable target giving a signal while intercalating in DNA. Accordingly, only when the bacterial plasma membrane become compromised, e.g., by an antimicrobial protein, fluorescence appears. The values were expressed as the mean of three independent experiments. Kinetics of the membrane permeabilization of *B. megaterium* were monitored in that fluorescence was measured for different doses at various time points and presented as a representative example from four replicates with similar results. The synthetic peptides alamethicin and cecropin P1 served as positive controls in the activity assays (Sigma, Taufkirchen, Germany).

## Results

### The multifarious Apl family

**Figure 1:**
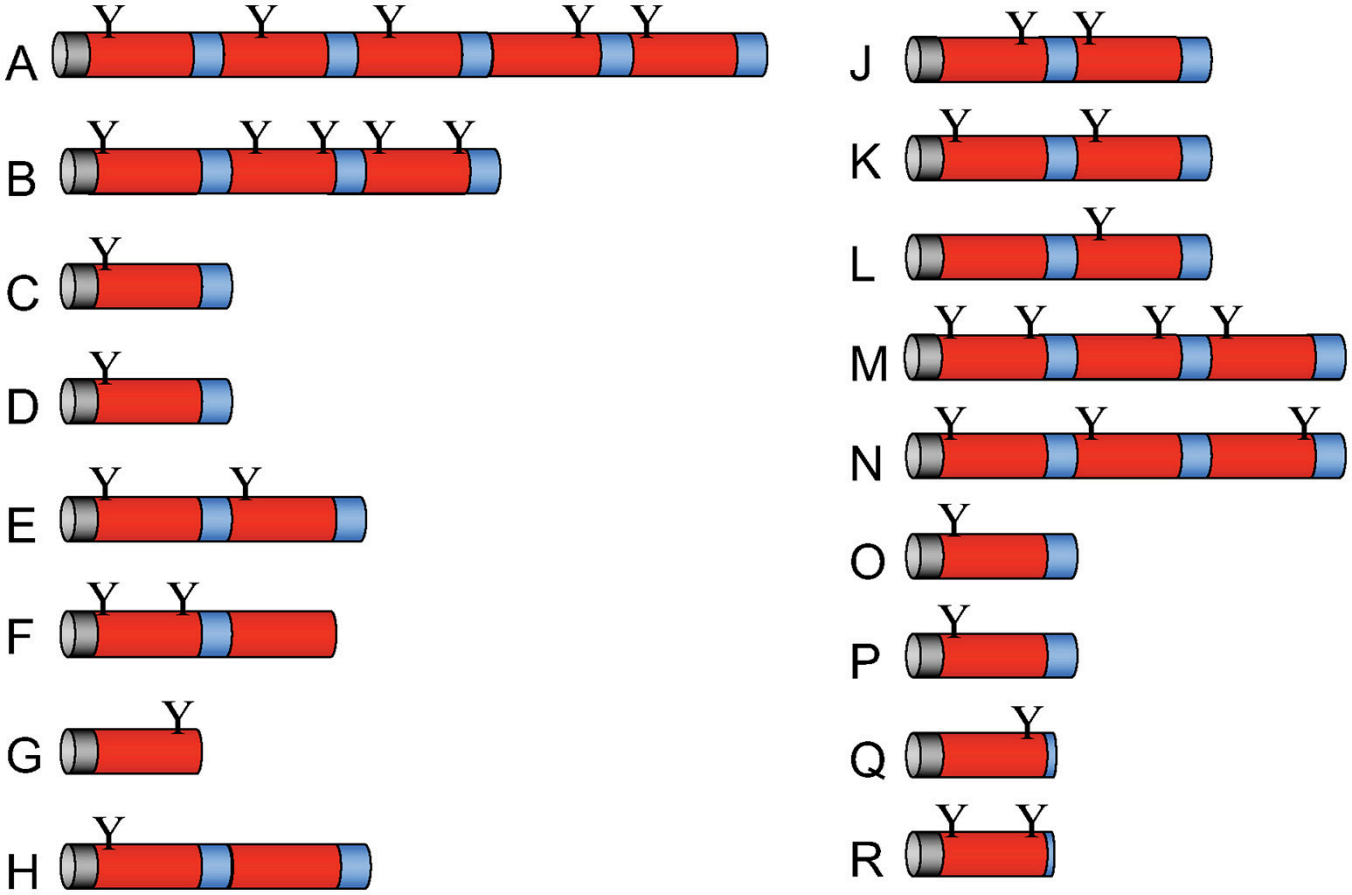
Molecular architecture of Apl precursor proteins. The *D. discoideum* genome contains 17 genes that potentially code for saposin-like proteins (SAPLIPs) with some similarity to amoebapores provisionally termed Apls. The primary translation products of *apl*s named from A to R (omitting I) contain between one and five SAPLIP domains (also termed saposin B domain). After processing, these preproproteins potentially give rise to 33 different mature polypeptides. Signal peptides (gray), SAPLIP domains (red), putative linker regions and end regions (blue), and potential N-glycosylation sites (Y) are depicted.

In the genome of *D. discoideum*, we detected 17 genes that may code for Apl proteins containing a single or several SAPLIP domains (Figure 1). The sequences of *apl* A-R and the corresponding putative proteins can be retrieved from the *D. discoideum* database (www.dictybase.org) using apl^*^ as a query. Their primary translation products each contain a predicted signal peptide that may allow trafficking of the precursor proteins to lysosomal compartments. Proteolytic processing of larger precursors can result in 33 mature peptides with one SAPLIP domain. An N-terminal glycosylation site can be predicted for nearly all of them.

### *Apl* mutants were defective for growth on virulent LM21 *K. pneumoniae*

**Figure 2:**
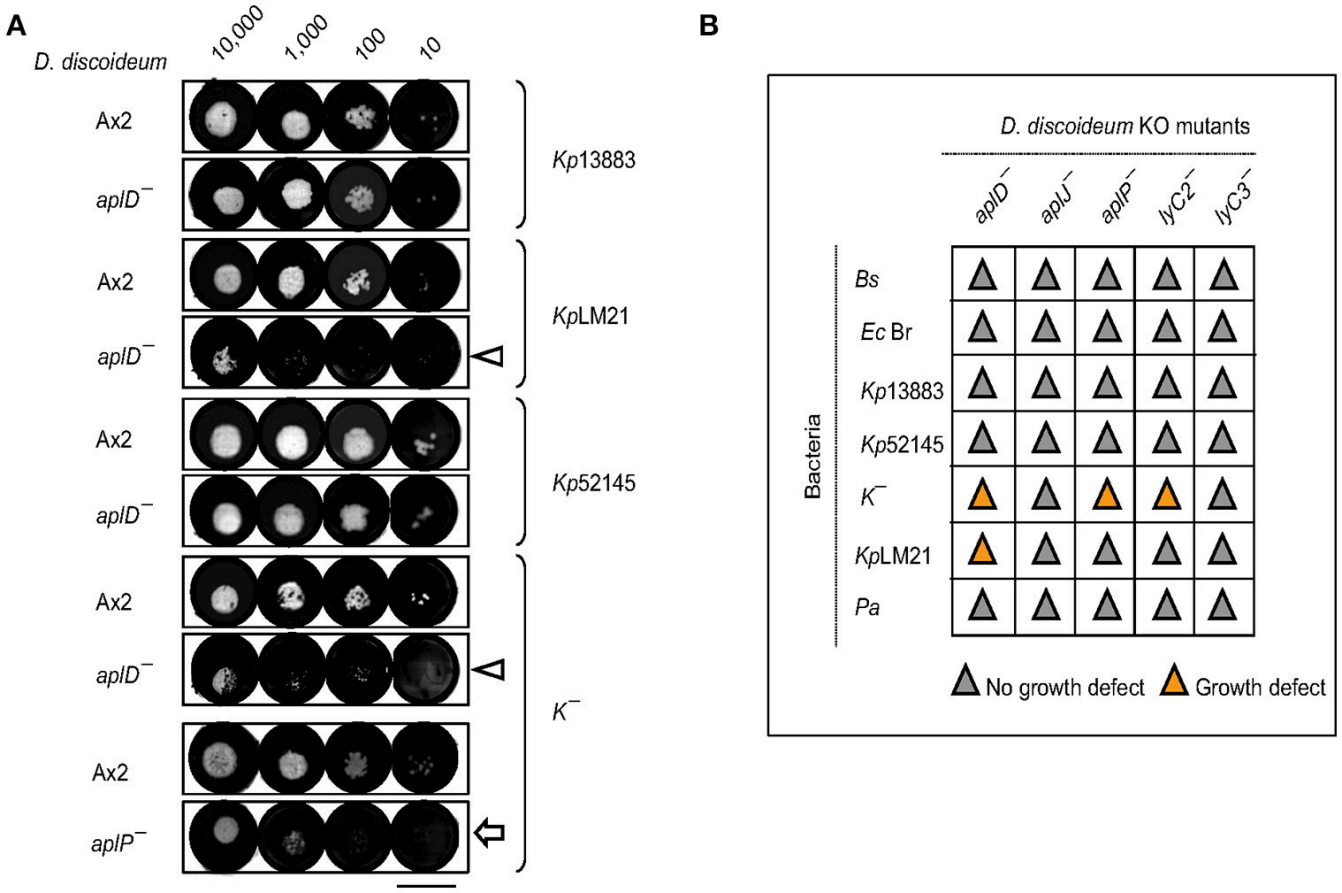
Growth defects of *AplD*^−^ and *aplP*^−^ cells on virulent *K. pneumoniae*. **(A)** *D. discoideum* growth abilities on various *K. pneumoniae* were tested by depositing indicated number of amoebae on *K. pneumoniae* lawns that were prepared on SM agar. *D. discoideum* amoebae were fed with *Kp*13883, *Kp*LM21, *Kp*52145, and *K*^−^. Arrowheads indicate *aplD*^−^ cells growth defects on *Kp*LM21 and *K*^−^. Arrow represents *aplP*^−^ growth impairment on *K*^−^. Scale bar, 1.5 cm. **(B)** Growth abilities of *aplD*^−^, *aplJ*^−^, *aplP*^−^, *lyC2*^−^ (see Supplementary Figure 5), and *lyC3*^−^ were tested on *B. subtilis* (*Bs*), *E. coli* B/r (*Ec* Br), *K. pneumoniae* strains (*Kp*13883, *Kp*52145, *K*^−^, and *Kp*LM21), and *P. aeruginosa* (*Pa*). Gray and yellow colors demonstrate the absence and presence of *D. discoideum* growth plaques on bacteria (see Supplementary Table 1 for bacterial strain details).

The permissiveness of various bacteria for amoebic growth is routinely assessed by testing the ability of *D. discoideum* mutants to generate growth plaques on bacteria lawns. This strategy has led to the identification of several bacterial virulence genes (Cosson et al., 2002; Pukatzki et al., 2002; Benghezal et al., 2007; Alibaud et al., 2008). Here, we adapted the method to test the growth abilities of *D. discoideum* Ax2 strain in which genes potentially coding for antimicrobial proteins (*apl*s and *lyC*s) have been disrupted. To quantitate the degree of bacterial virulence, varying number of *D. discoideum* cells were spotted on bacterial lawns and the growth plaques were monitored after 5 days. It became apparent that *aplD*^−^ was defective for growth on *Kp*LM21, a clinical isolate and two *apl* mutants, *aplD*^−^ and *aplP*^−^, were non-permissive for growth on *K*^−^, a capsule defective *Kp* strain (Figure 2A). In the case of Ax2, even 10 amoebae were sufficient to create growth plaques on *K*^−^ and *Kp*LM21, whereas at least 10,000 amoebae of *aplD*^−^ and at least thousand amoebae of *aplP*^−^ were required to generate visible plaques on *K*^−^. Likewise, *Kp*LM21 allowed plaque formation only when 10,000 *aplD*^−^ amoebae were deposited (Figure 2A). We also found that *D. discoideum* with a KO of the gene encoding the C-type lysozyme 2, *lyC2*^−^, was defective for growth on *K*^−^ (Supplementary Figure 5). The growth abilities of *Apl*s and *lyC*s mutants on bacteria such as *B. subtilis, E. coli Br*, non-pathogenic *K. pneumoniae*, and *P. aeruginosa* are summarized in a scheme (Figure 2B).

### Vegetative *aplD^−^* amoebae efficiently kill *K. pneumoniae Kp*LM21

**Figure 3:**
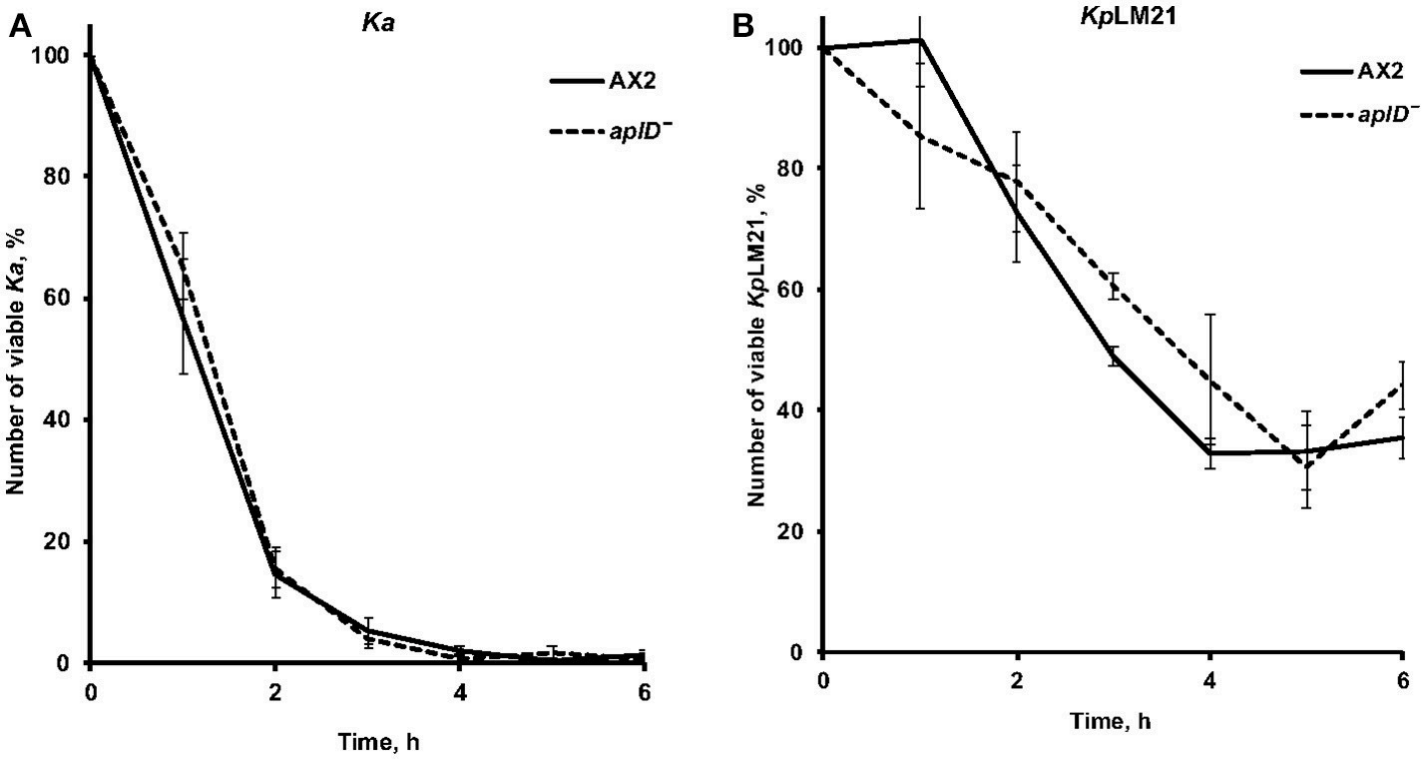
*AplD*^−^ cells kill efficiently *Klebsiella pneumoniae*. **(A)** The ability of *D. discoideum* to ingest and kill non-virulent *K. aerogenes Ka* was tested by mixing amoebae and bacteria and measuring the total number of viable bacteria at the indicated time points. **(B)** The same assay was performed using virulent *K. pneumoniae Kp*LM21. Ax2 was used as comparative control. Each curve is the average of three independent experiments, bars represent standard error of the mean.

As described in Benghezal et al. (2006), we performed a specific assay to investigate whether *aplD*^−^ amoebae are defective in phagocytosis and/or killing of *Kp*LM21. These assays measure the total number of live bacteria remaining at indicated time points. We found that *aplD*^−^ amoebae were able to phagocytose and kill *Klebsiella aerogenes* and *Kp*LM21 as efficiently as the wild-type Ax2 (Figure 3). This indicates that AplD is not essential for intracellular killing of *K. pneumoniae* bacteria in free-living *Dictyostelium* amoebae.

### *AplD* upregulation upon exposure to *Kp*LM21

**Figure 4:**
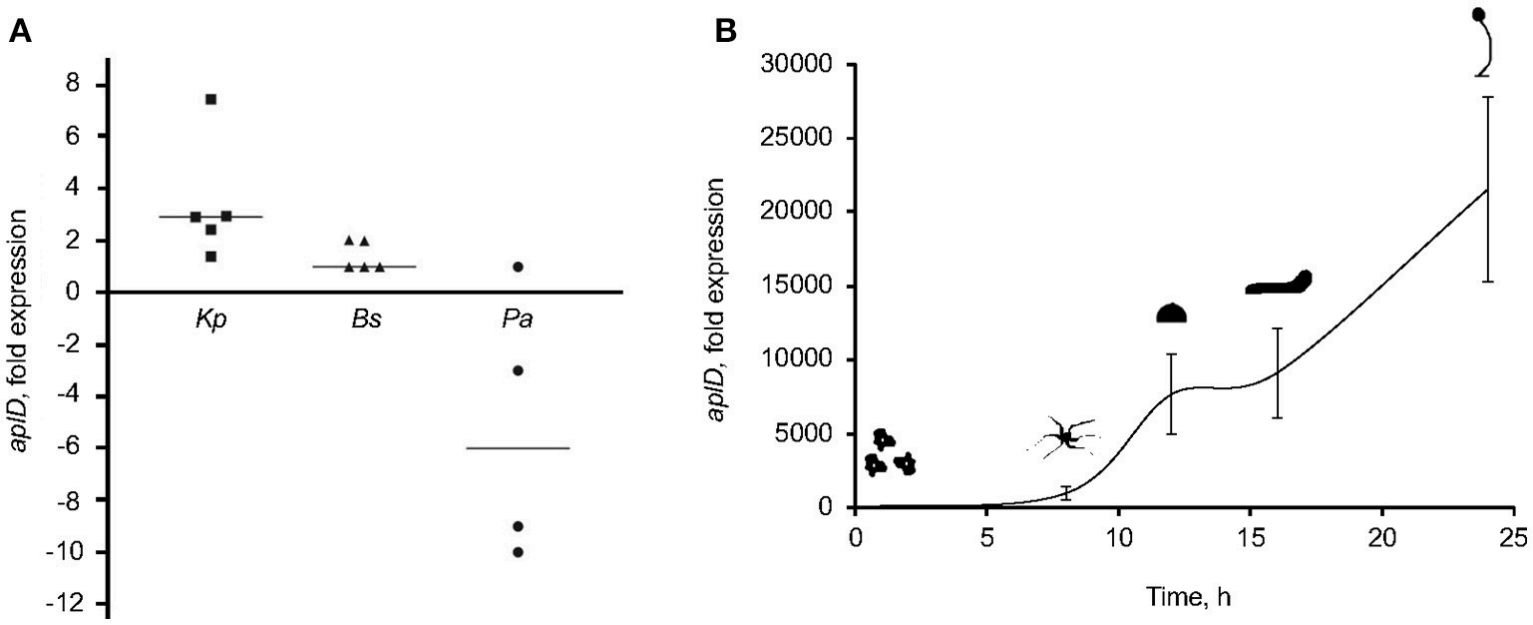
*AplD* upregulation upon *K. pneumoniae* feeding and during late development. **(A)** *AplD* transcription profile in xenic Ax2 cultures were examined by mixing amoebae with bacteria at an 1:10 ratio and measuring *aplD* transcript levels by qRT-PCR. *AplD* fold expression in xenic Ax2 cultures were determined based on *aplD* transcription in axenic Ax2 cultures. Bacterial strains tested were *K. pneumoniae* (*Kp*LM21), *B. subtilis* (*Bs*), and *P. aeruginosa* (*Pa*). Each symbol denotes the data from an individual experiment and the bar represents the median thereof. **(B)** *AplD* regulation during Ax2 development was tested by subjecting Ax2 for development on KK2 agar plates and quantifying *aplD* transcription at different morphogenetic stages by qRT-PCR. *AplD* expression at 0 h was considered as reference to determine *aplD* fold expression at subsequent development stages. *n* = 3, bars represent standard deviation.

To examine whether the expression of *aplD* is regulated by exposure to various bacteria, we measured the level of the *aplD* mRNA by qRT-PCR. Contact with *Kp*LM21 increased *aplD* at least three-fold, but contact with *Bacillus subtilis* did not induce a significant change. In contrast, exposure to *Pseudomonas aeruginosa* PT531 downregulated *aplD* on average about 6-fold (Figure 4A). Analyses of *aplD* regulation during *D. discoideum* development on non-nutrient KK2 agar showed that *aplD* was weakly expressed in axenic conditions, but was strongly upregulated during late development stages, starting from the mound stage [12 h] and slug stage [16 h], and peaking at fruiting body stage [24 h] (Figure 4B). This profile is comparable to the *aplD* transcriptional regulation observed in Ax4 grown on bacteria and subjected to development on SB agar (Dicty express, Supplementary Figure 6). The transcriptional regulation of antimicrobial genes during the growth phase of *D. discoideum* might be essential to adapt to various food sources, but also combat various pathogens. It has been reported that amoebae stop feeding during development. Therefore, the main purpose for the *aplD* upregulation might be an extracellular role in cellular defenses inside the multicellular structures or inside phagocytic Sentinel cells. Interestingly, upregulation of *aplD* during development, in particular at late stages, is several folds higher than during exposure to *Kp*LM21.

### *AplD^−^* slugs are vulnerable to *K. pneumoniae*

**Figure 5:**
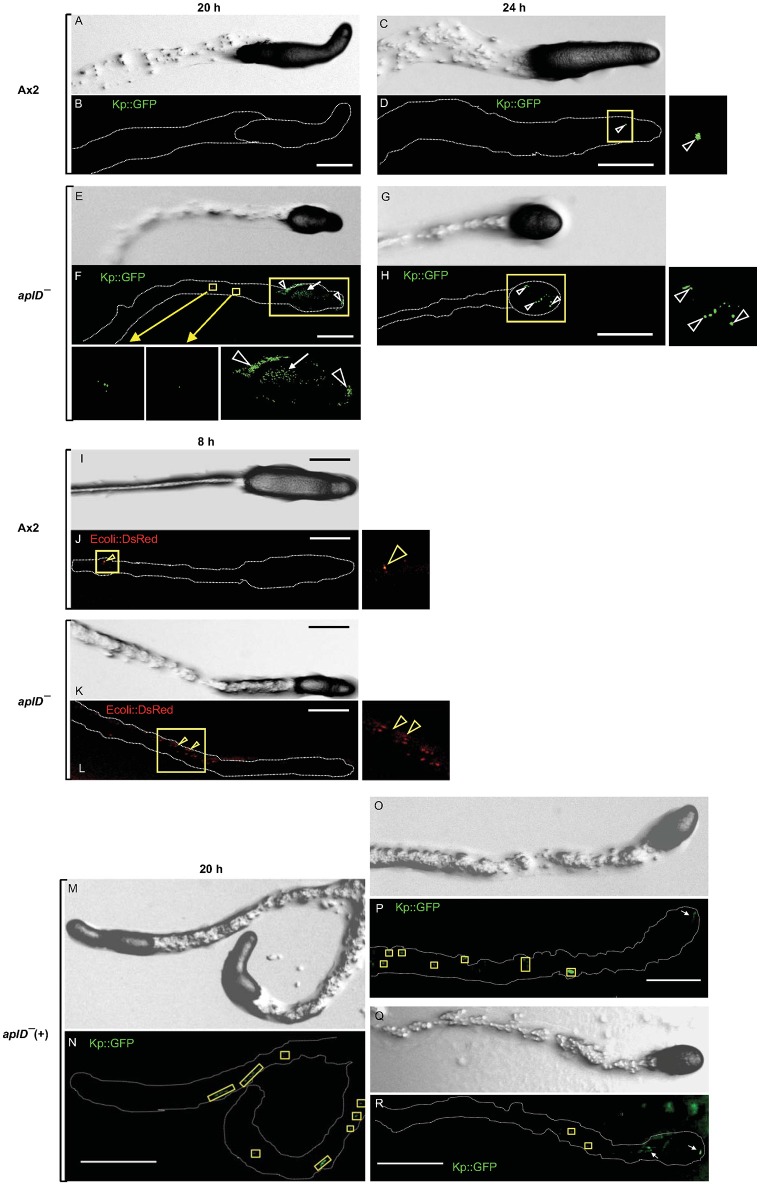
Susceptibility of slugs to infection with *Klebsiella* and *E. coli*. Migrating *D. discoideum* slugs were injured with a fine needle and infected with GFP-expressing *K. pneumoniae* (*Kp*GFP). Slugs were imaged at 20 and 24 h post *Kp*GFP infection. Differential Interference Contrast (DIC) images of Ax2 **(A,C)** and *aplD*^−^ slugs **(E,G)** at 20 and 24 h post infection are represented. *Kp*GFP infections in Ax2 **(B,D)** and *aplD*^−^ slugs **(F,H)** at 20 and 24 h post infection are shown under green fluorescence filter. *Kp*GFP infection in Ax2 slug imaged 24 h post *Kp*GFP infection **(D)** is also magnified. *Kp*GFP infection on slug surface (arrowheads) and slug interior (arrow) and in the slime sheath of *aplD*^−^ slug at 20 h post infection are magnified in the insets below. *Kp*GFP clumps inside *aplD*^−^ slug 24 h post infection **(H)** is magnified in the adjacent panel. *AplD*^−^ slugs sloughed off *E. coli* in their slime sheaths. DIC images of Ax2 and *aplD*^−^ slugs **(I,K)** 8 h post infection with *E. coli* DsRed are represented. *E. coli* DsRed clumps (arrowheads) in Ax2 **(J)** and *aplD*^−^ **(L)** slime sheaths (boxes) are shown under red fluorescence filter and are also magnified in the respective insets. *AplD*^*-*^(+) slugs sloughed off *Kp*GFP in their slime sheaths. *AplD*^−^(+) slugs were infected with *Kp*GFP as described earlier and were imaged 20 h post *Kp*GFP infection. DIC images of *aplD*^−^(+) slugs are shown in **(M,O,Q)**. *K. pneumoniae* infections in *aplD*^−^(+) slugs are shown under green fluorescence filter in **(N,P,R)**. *AplD*^−^(+) slime sheaths showing *Kp*GFP deposits (boxes) and clumps in the slug interior (arrows) at 20 h post infection are depicted. Scale bars, 100 μm.

**Figure 6:**
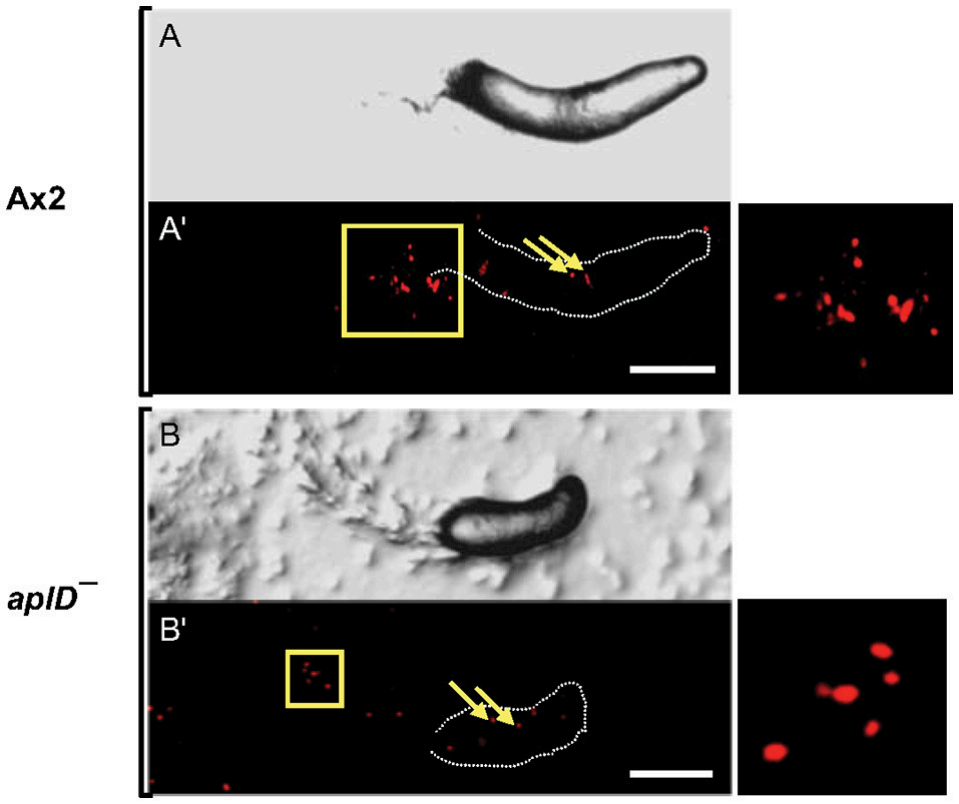
Sentinel cells were unaffected in *aplD*^−^ slugs. Ax2 and *aplD*^−^ slugs were allowed to migrate on EtBr-containing (3 μg/ml) agar for 3 h. DIC images of Ax2 and *aplD*^−^ slugs **(A,B)** imaged under a stereomicroscope are represented. Sentinel cells inside Ax2 and *aplD*^−^ slugs **(A',B')** are shown under red fluorescence filter (arrows). Sentinel cells detected in slime sheaths are magnified (boxes, right). Scale bars, 100 μm.

To test the idea that AplD is primarily instrumental in multicellular stages of *D. discoideum*, we infected slugs derived from *aplD*^−^ amoebae with a *K. pneumoniae* (*Kp*) strain that expressed GFP. Unlike wild type slugs, at 20 h post *Kp* infection, *aplD*^-^ slugs showed bacterial deposits both on their surface and in their body. At 24 h post *Kp* infection, *aplD*^−^ slugs showed *Kp* clumps mainly inside. Additionally, *aplD*^−^ slugs were not successful in sloughing off *Kp* in their slime sheath (Figure 5). By contrast, *aplD*^−^ slugs were able to shed *E. coli* in their slime sheath as early as 8 h post infection (Figure 5). As the major defense mechanism reported so far in *D. discoideum* slugs is based on the phagocytic nature of Sentinel cells, we examined whether *aplD*^−^ slugs possess a functional population of these cells. As evidenced by the uptake of the fluid-phase tracer EtBr, *aplD*^*-*^ slugs were capable of generating Sentinel cells that internalized the toxic dye and were sloughed off during migration (Figure 6) and *aplD*^−^ did not depict any prominent developmental defect (Supplementary Figure 4).

### *Klebsiella pneumoniae* susceptibility was rescued in *aplD^−^*(+) amoebae and slugs

**Figure 7:**
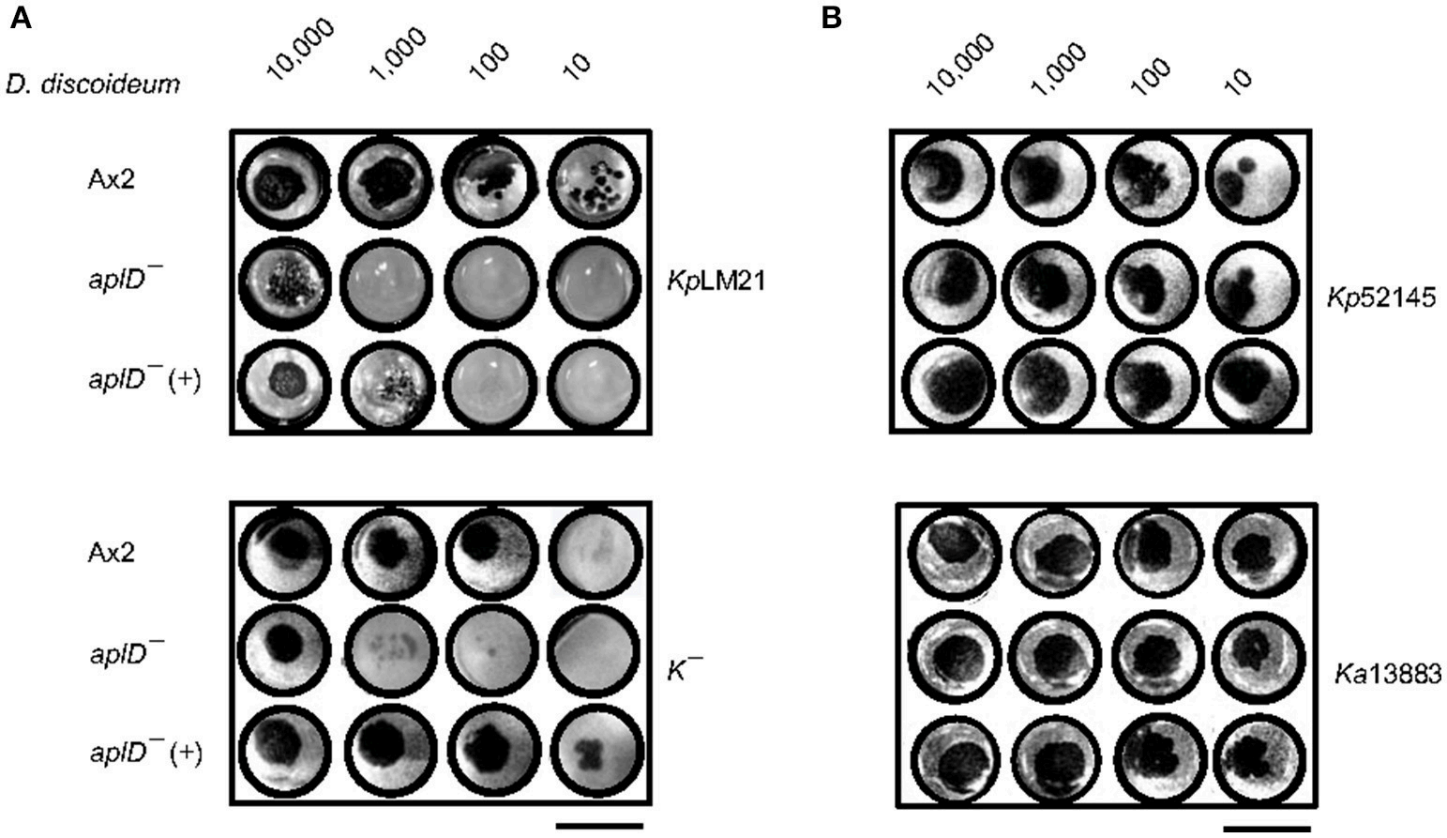
*AplD*^−^(+) strain growth on *K. pneumoniae*. Growth abilities of *aplD*^−^(+) cells were tested on *K. pneumoniae Kp*LM21 and *K*^−^ **(A)** and on *K. pneumoniae Kp52145* and *Ka* 13883 **(B)**. Growth abilities of Ax2 and *aplD*^−^ cells are also represented for comparison. Scale bar, 1.5 cm.

As described above, *aplD*^−^ amoebae were restricted for growth on *K. pneumoniae, K*^−^, and *Kp*LM21, and *aplD*^*-*^ slugs were vulnerable to *Kp* infections. We tested whether restoration of *aplD* expression in *aplD*^−^ would rescue the phenotypic defects. Constitutive expression of *aplD* in *aplD*^−^ amoebae, termed *aplD*^−^(+), under the control of the *act6* promoter of an extra-chromosomal vector rescued the formation of plaques on *K*^−^ but showed only partial rescue on *Kp*LM21 lawns (Figure 7). Bacterial clearing was observed on *Kp*LM21 lawns when 10^4^ and 10^3^ amoebae were deposited, but not with 10^2^ and 10^1^ amoebae. This observation might indicate that spatio-temporal regulation of *aplD* expression is crucial for full functionality. *AplD*^−^(+) slugs infected with *Kp* were able to clear off *Kp* by sloughing them off in their slime sheath. It appeared that the interior of *aplD*^−^(+) slugs was virtually free of *Kp* deposits and clumps (Figure 5).

### *In-vitro* functional analysis of the protein AplD

**Figure 8:**
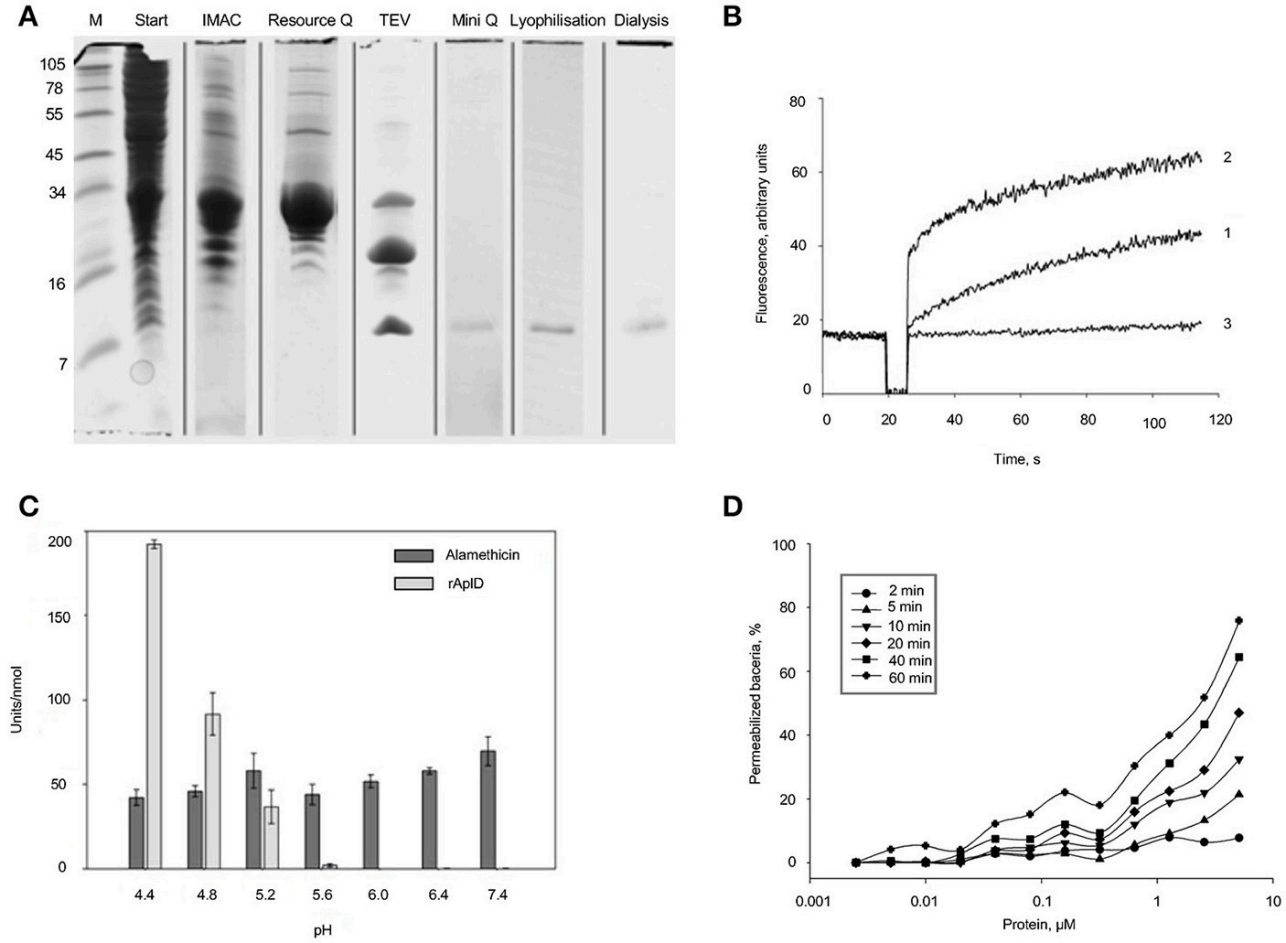
Purification and functional *in-vitro* analysis of recombinant AplD. **(A)** Purification steps of AplD after recombinant synthesis evidenced by SDS PAGE and Coomassie staining of the gel: Extracts of *E. coli* C43 transformed with *aplD* in pET-32a (+) in TBS, pH 7.0 (start), enrichment with IMAC, further purification with anion-exchange chromatography (Resource Q), proteolytic cleavage with TEV, and final purification via a Mini Q column. Purity of AplD after lyophylisation and dialysis is also shown at the right. All samples were reduced and alkylated before separation. At the very left, marker proteins (M) were separated and the molecular masses of these are depicted in kDa. **(B)** Time course of pore formation induced by AplD. The dissipation of a valinomycin-induced diffusion potential in vesicles of asolectin after addition of AplD (80 pmol) (trace 1), control peptide alamethicin (100 pmol) (trace 2), and the peptide solvent (trace 3) were recorded at pH 5.2. Pore-forming activity is reflected by the increase of fluorescence as a function of time. **(C)** pH dependence of pore-forming activity of AplD. Activity of AplD in comparison with that of alamethicin was measured in six independent experiments in Tris-maleate buffer (50 mM Tris-maleate/50 mM sodium sulfate/0.5 mM EDTA/0.02 % sodium azide) adjusted to various pH. Mean and standard deviation are shown. **(D)** Kinetics of membrane permeabilization by AplD. Membrane permeabilization of viable *B. megaterium* was measured as an increase in fluorescence of the DNA intercalating dye SYTOX Green at pH 5.2 induced by AplD and monitored after different time points at various concentrations.

AplD was heterologously synthesized in *E. coli* as a fusion protein and subsequently the thioredoxin-His_6_ tag was removed by proteolytic cleavage. The final product was purified to apparent homogeneity by repeated steps of IMAC and anion-exchange chromatography (Figure 8A). Analysis by MALDI-TOF mass spectrometry confirmed the molecular identity of rAplD. It revealed an experimental average mass of 9,070.8 Da, which is in good agreement with the calculated molecular mass (9,070.3 Da) provided that six cysteine residues are involved in disulfide bonds. The recombinant protein was tested for its pore-forming and bacterial-membrane permeabilizing activities *in vitro*. AplD formed pores in liposomes composed of asolectin. At mildly acidic pH (5.2), AplD depolarized liposomal membranes with similar activity as the prototype of a pore-forming peptide, alamethicin (Figure 8B). AplD acted in a pH-dependent manner. The protein displayed the highest pore-forming activity at pH 4.4 and gave decreasing values with increasing pH (Figure 8C). In the Sytox-Green assay, performed exemplarily with *B. megaterium*, it became apparent that AplD is capable of permeabilizing the cytoplasmic membranes of live bacteria. Membrane-permeabilizing activity increased with time. After 2 min, virtually no activity was detectable, but already after 5 min bacteria with compromised membranes appeared, particularly at higher protein concentrations. After 1 h, approximately 50% of the bacteria were permeabilized by AplD at 2.5 μM (Figure 8D). We could not detect bacterial membrane permeabilization with up to 5 μM AplD in the same assay when we used Gram-negative bacteria, *K. pneumoniae* LM21, *K. pneumoniae K*^−^, *K. aerogenes*, or *E. coli*, as target cells (data not shown) indicating that the outer membrane of these species constitutes an additional barrier for AplD for reaching the bacterial cytoplasmic membrane.

## Discussion

*Dictyostelium discoideum* is a non-pathogenic, unicellular host used for bacterial and fungal risk assessment studies (Cosson et al., 2002; Alibaud et al., 2008; Koller et al., 2016) and the outcomes from such studies remain surprisingly similar to the observations made in animal hosts (Benghezal et al., 2006; Hagedorn et al., 2009). Although *D. discoideum* serves as a versatile system to explore bacterial virulence genes and their mechanisms of action, not much is known about the effector molecules of *D. discoideum* and their mode of action to defend against pathogens. In *D. discoideum*, host-pathogen interaction studies can be attempted both at the single-cell phagocyte stage and during the multicellular stages of its life cycle. Interestingly, the single-cell and multicellular phases are clearly separated and involve differential regulation of gene expression. During the growth phase, *D. discoideum* internalizes microbes by phagocytosis and the resulting phagosome fuses with lysosome, a compartment that houses hydrolytic enzymes and antimicrobial peptides, leading to efficient killing and degradation of the microbes to fulfill the nutritional need of the growing amoeba.

However, several intracellular pathogens have evolved mechanisms to block phago-lysosome fusion and to exploit the phagosomal compartment as their replication hub (Hagedorn et al., 2009; Shevchuk et al., 2014). During the multicellular stages of development, the antibacterial defense mechanisms are even more sophisticated. For example, in the slug formed mainly of non-phagocytic prespore and prestalk cells, about 1% of the cells, called the Sentinel cells retain their phagocytic ability. The foreign bodies and poisonous compounds that enter the slug are swallowed by these cells and finally get sloughed off the migrating slug and remain in the slime sheath, where they are entangled within extracellular DNA traps (Zhang and Soldati, 2016; Zhang et al., 2016). Mechanistically, this helps to restrict microbial infection and/or the accumulation of toxins. The present study reveals for the first time the specific involvement of the AplD gene product in antimicrobial defense during multicellular stages of development. In addition, *aplD* expression is differentially regulated by exposure to different bacteria, e.g., induced in the presence of *K. pneumoniae* and repressed in the presence of *P. aeruginosa*.

These *in vivo* evidences strongly suggest that Apl peptides are involved in antimicrobial defense, but a direct membrane-lysing activity had not been reported so far. Here, we show that purified, recombinant AplD displayed pore-forming activity in the minimalistic system of liposomes and also readily permeabilized the membranes of the live Gram-positive bacterium *B. megaterium* as monitored by the Sytox green assay. However, an activity against Gram-negative bacteria was not detected at the concentrations employed. One might speculate that AplD can also permeabilize *Klebsiella*, provided that the outer membrane does not hamper the access to the primary target, the bacterial plasma cell membrane. The antimicrobial efficacy of the relatively negatively charged AplD might be substantially enhanced by the synergistic action of other antimicrobial proteins, e.g., Apls with a more positive net charge, or when other *Dictyostelium* factors that are not bactericidal *per se* but are capable of perturbing the outer membrane structure act in concert with AplD.

There are several examples of SAPLIPs including the saposins themselves that have been found to be glycosylated in their natural form. The vast majority of the potential peptides released from Apl precursors bear a potential N-glycosylation site. This also holds true for AplD. In amoebae, we have previously compared the pore-forming activity toward liposomes of glycosylated and deglycosylated naegleriapores and could not detect a substantial negative effect upon glycan removal (Herbst et al., 2002). However, we cannot exclude that a natural AplD, glycosylated specifically by *D. discoideum*, might have a different activity spectrum against natural targets than the recombinant unglycosylated version. A single glycosylation motif may determine a particular oligomeric structure, e.g., dimerization, to generate a more active protein, it may mediate an interaction with a partner molecule that helps to overcome the outer membrane of Gram-negative bacteria such as *Klebsiella*, or it may simply facilitate binding to bacterial targets, e.g., by shielding the negative charge of particular regions of the protein.

Apart from Apls, there are two other proteins that harbor a SAPLIP domain, i.e., acyloxyacyl hydrolase (AOAH) and countin (Ctn). The enzyme AOAH deacylates lipopolysaccharides in vertebrates, but has not been characterized in amoebae so far (Munford et al., 2009). Countin is a well-studied major component of a protein complex called counting factor, known to play a crucial role in aggregate size determination during development (Brock and Gomer, 1999). When recombinant countin was monitored for pore-forming activity in a liposome-depolarization assay, it exhibited only marginal activity (Gao et al., 2002). In our study, we found that *aplD*^−^ amoebae showed mild defects at early development, such as stream breaks and developmental delay, but finally were able to form slugs and fruiting bodies, although they were smaller than from wild-type Ax2 cells.

We found that *aplD*^−^ amoebae are specifically impaired for growth on one virulent strain of *K. pneumoniae*. Although *apl*D is poorly transcribed in the amoebic stage under axenic growth conditions, it appears as if its gene product is nevertheless instrumental to allow growth in the presence of some *K. pneumoniae* strains. The plethora of *Klebsiella* bacteria present over a longer period in the growth assay, different from the short exposure to *K. pneumoniae* in a killing assay, may change gene expression of several genes including *apl*D. More impressively, we found that *aplD*^−^ slugs are immune-compromised specifically for *K. pneumoniae*. During that multicellular stage, we observed a dramatic increase of *aplD* transcript abundance. Notably, complementation of *aplD* in *aplD*^−^ rescued the capacity of slugs to effectively capture and slough off *K. pneumoniae* in their slime sheath in a way similar to wild-type Ax2.

With respect to the evolution of innate immunity, the antimicrobial capacities of *D. discoideum* are still a relatively unexplored area of research. On the one hand, the amoeba is an interesting model because it resembles in its unicellular stage mammalian macrophages in several aspects (Bozzaro et al., 2008; Cosson and Soldati, 2008; Dunn et al., 2018). On the other hand, the developmental cycle of the social amoeba represents an intriguing example in biology of appearance of multicellularity before the advent of true metazoans. In particular, the slug, which is composed of about 100,000 prespore and prestalk cells, hosts about 1% of Sentinel cells. These phagocytic cells reminiscent of patrolling neutrophils and tissue macrophages of animals, represent an extraordinarily appealing model system for comparative immunologists, as they defend the slug against infection using bactericidal phagocytosis and DNA extracellular traps (Chen et al., 2007; Zhang et al., 2016). Here, we reveal the involvement of an amoebic SAPLIP in the struggle of the slug stage against virulent bacteria, as an early example of a molecular defense implemented at the transition from unicellular to multicellular organisms.

## Author contributions

Conceived the study: ML. Designed the experiments: RD, PC, TS, and ML. Performed the experiments: RD and MB. Analyzed the data: RD, MB, PC, TS, and ML. Wrote the paper: RD, PC, TS, and ML.

### Conflict of interest statement

The authors declare that the research was conducted in the absence of any commercial or financial relationships that could be construed as a potential conflict of interest.
