# Supplementary material for: The Saposin-Like Protein AplD Displays Pore-Forming Activity and Participates in Defense Against Bacterial Infection During a Multicellular Stage of Dictyostelium discoideum
Source: Front Cell Infect Microbiol. 2018 Mar 15;8:73. doi: 10.3389/fcimb.2018.00073 (PMC5890168; doi:10.3389/fcimb.2018.00073)
Supplement: Supplementary file 1 [file Presentation1.pdf]

The saposin-like protein AplD displays pore-forming activity and participates in defence against bacterial infection during a multicellular state of *Dictyostelium discoideum*

Supplementary Figure 1

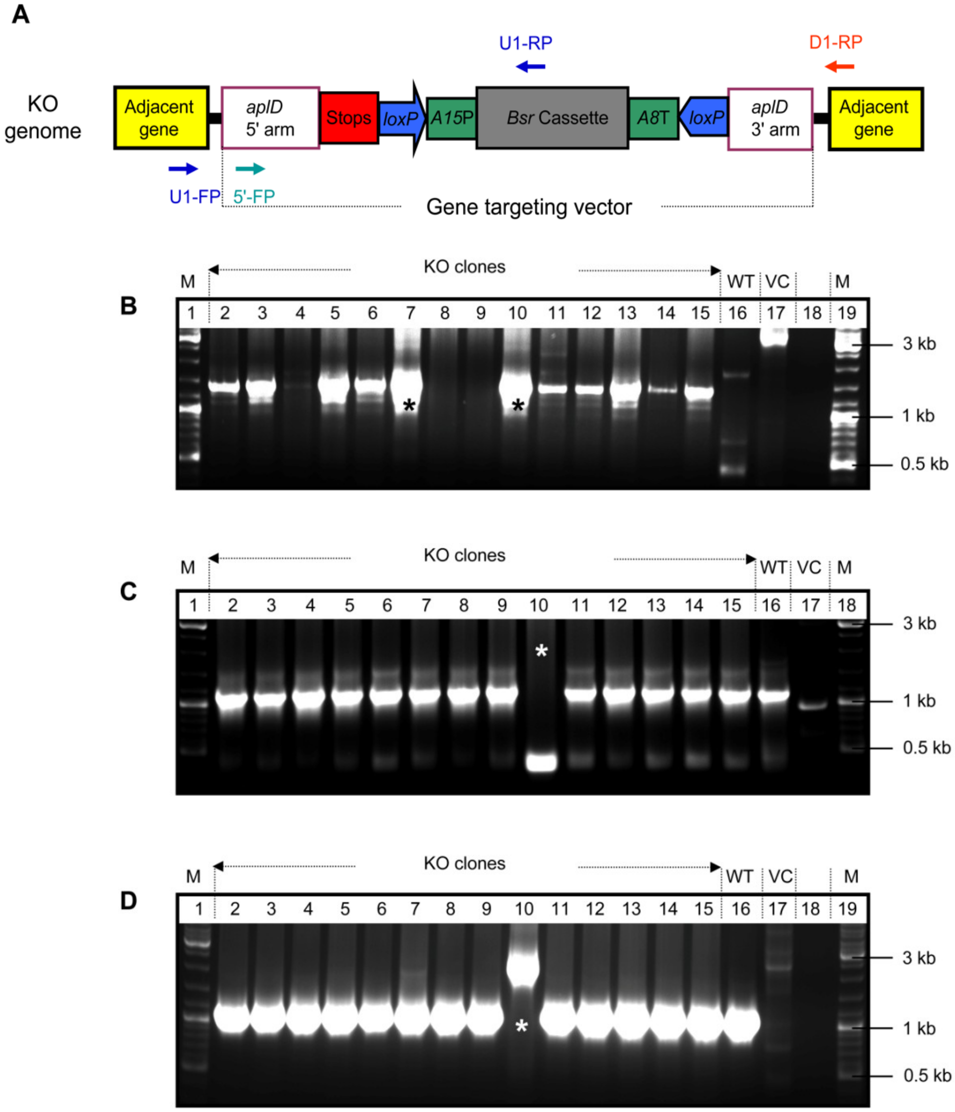

Supplementary Figure 1: PCR analyses of *aplD*⁻ clones. (A) PCR screening strategies followed to examine *aplD*⁻ clones. Arrows represent primers used for PCR analyses. (B) *AplD*⁻ clones PCR analysis using U1-FP and U1-RP primers. Ax2 genomic DNA (wildtype; WT) and *aplD* KO vector (vector control; VC) were used as positive/negative controls. Recombinants generated 1.4 kb amplicon (lane 7* and 10*), WT and VC produced no amplicon. (C) *AplD*⁻ clones PCR analysis with U1-FP and D1-RP primers. All clones produced positive signal for WT (1.1kb) except one clone (lane 10*). (D) *AplD*⁻ clones PCR analysis using 5'-FP and D1-RP primers. Recombinant clone (lane 10*) generated 1.3 kb larger amplicon than the WT. In C and D, WT was considered as positive control and VC as negative control. Asterisk symbols denote positive clones for *aplD* ablation. Primers: U1-FP: 5'-AGTGAAGAAATTGTGGGTGTGTG-3'; U1-RP: 5'-CGCTTCAATATGTACTGCCGAAATG-3' ; D1-FP: 5'-GCAAACAATTTGTTGATTCAAATTTCC-3'; D1-RP: 5'- GTCGACGTAATGAATTGTTTAAAACTCG-3' SalI; 5'- FP: 5'- GGGAGTACTTTGAAACCACC-3'.

Supplementary Figure 2

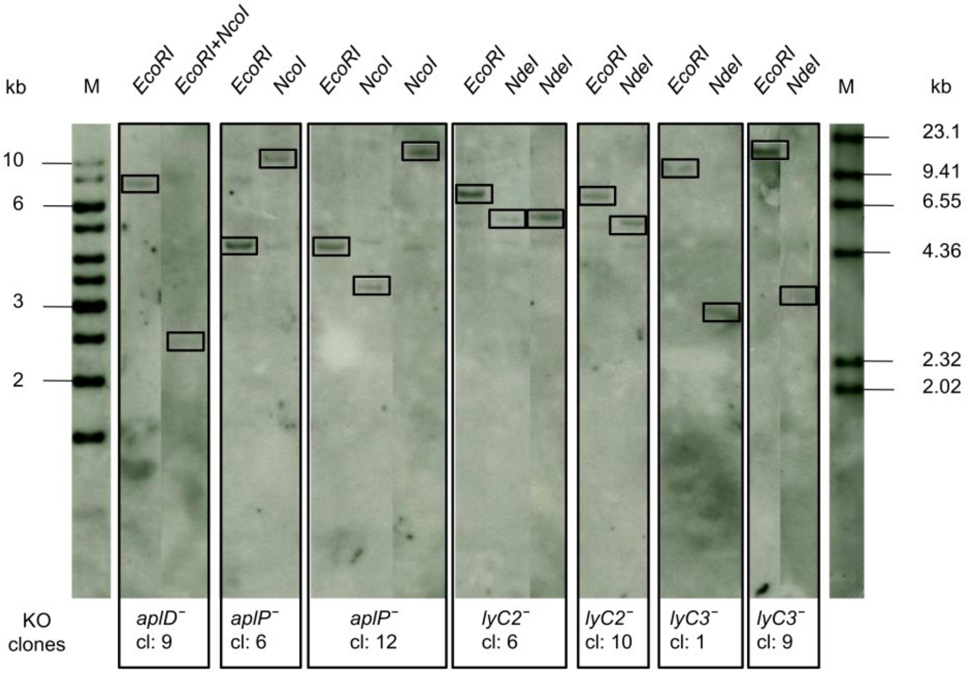

Supplementary Figure 2: Southern hybridization confirming *apl* and *lyC* KOs. Genomic DNA was isolated from the KO clones and subjected to restriction endonuclease digestions. Fragmented genomic DNA samples were separated on agarose gels, Bsr and gene specific probes were employed to confirm KO vector insertions at respective loci in *D. discoideum* genome. KO clones examined include *aplD⁻*, *aplP⁻*, *lyC2⁻*, and *lyC3⁻*. M represents DNA markers. Two independent clones were examined for each KO except for *aplD*.

Supplementary Figure 3

A

B

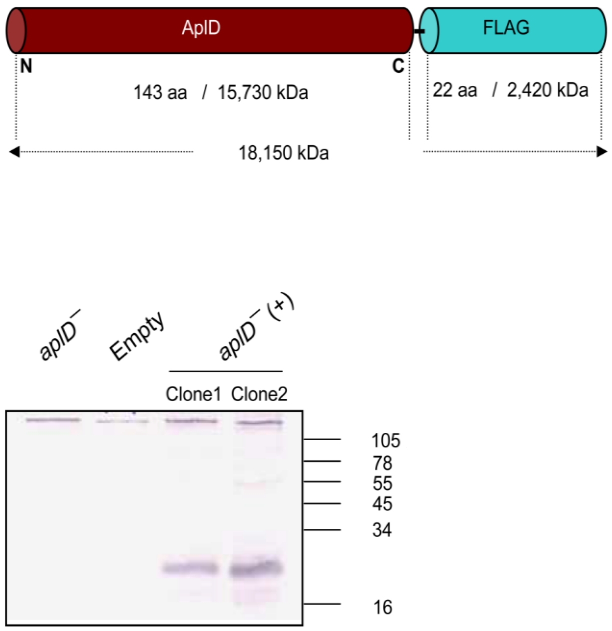

Supplementary Figure 3: Architecture and identification of AplD-FLAG tag protein produced in rescue strain. (**A**) AplD-FLAG tag protein: FLAG tag was fused at 3' region of *aplD* cDNA to produce AplD protein with C-terminal FLAG tag. (**B**) Western blot analysis: Cell lysates were prepared from *apllD*$^{-}$ cells (control) and *aplD*$^{-}$/[act6]:*aplD*.FLAG cells [rescue strain; mentioned as *aplD*$^{-}$(+)]. Two clones of the rescue strain were positive for AplD-FLAG fusion protein synthesis and no signal was observed in *aplD*$^{-}$ cells. Molecular masses in kDa are shown at the right.

Supplementary Figure 4

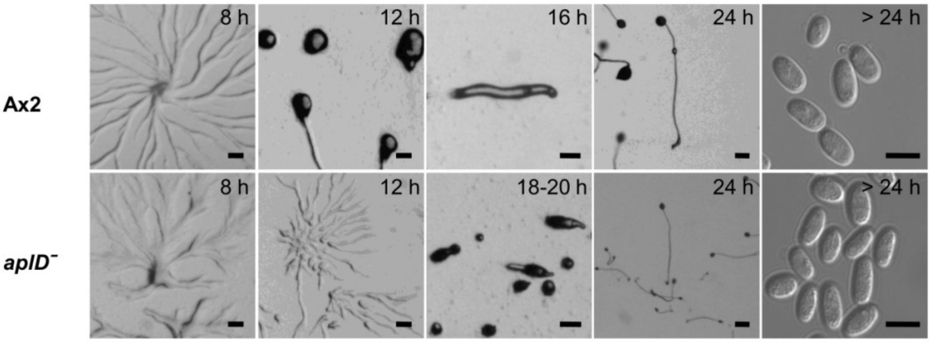

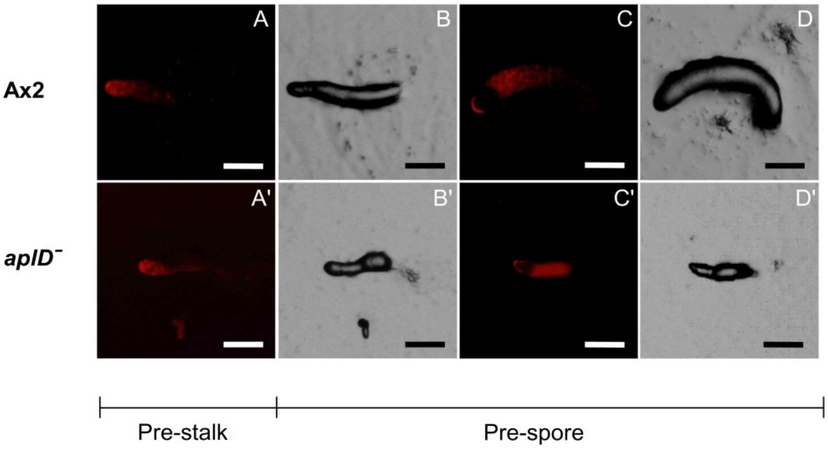

Supplementary Figure 4: Delayed and aberrant streaming patterns of *aplD*⁻ cells (top panel). *D. discoideum* (Ax2 and *aplD*⁻) morphogenic structures were imaged under stereomicroscope at indicated time points. Scale bars, 10 µm (spores) and 100 µm. Pre-stalk and pre-spore slug patterns unaffected in *aplD*⁻ slugs (bottom panel). *D. discoideum* cells carrying pre-stalk (*ecmAO*::RFP) and pre-spore (*pspA*::RFP) markers were mixed with unlabeled *D. discoideum* cells and allowed to develop. After 20 h, Ax2 (**A** and **B**) and *aplD*⁻ slugs (**A'** and **B'**) expressing *ecmAO*::RFP marker were imaged under red fluorescence and DIC filters. Ax2 (**C** and **D**) and *aplD*⁻ slugs (**C'** and **D'**) depicting *pspA*::RFP marker expression are also shown. Horizontal line denotes slug orientations. Scale bars, 100 µm

Supplementary Figure 5

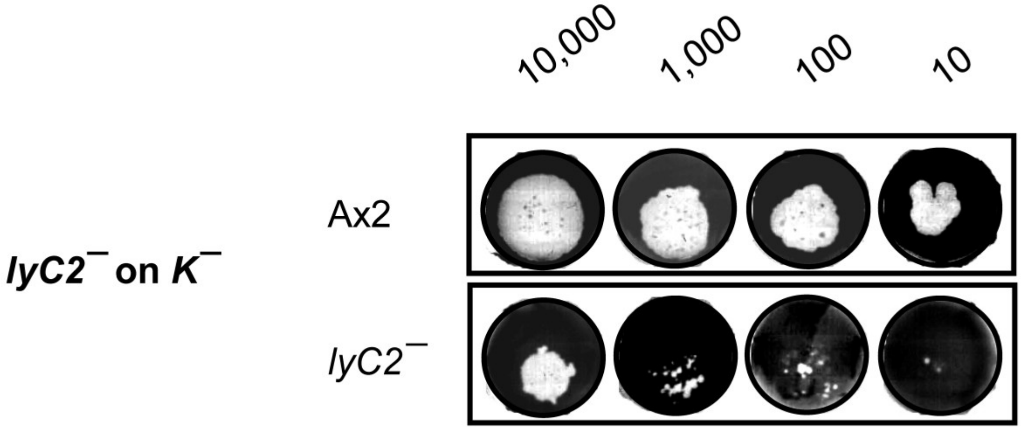

Supplementary Figure 5: Varying numbers of *lyC2⁻* amoebae were spotted on the overnight culture of *K⁻* that was uniformly layered on SM agar. Ax2 growth on *K⁻* is also shown for comparison

Supplementary Figure 6

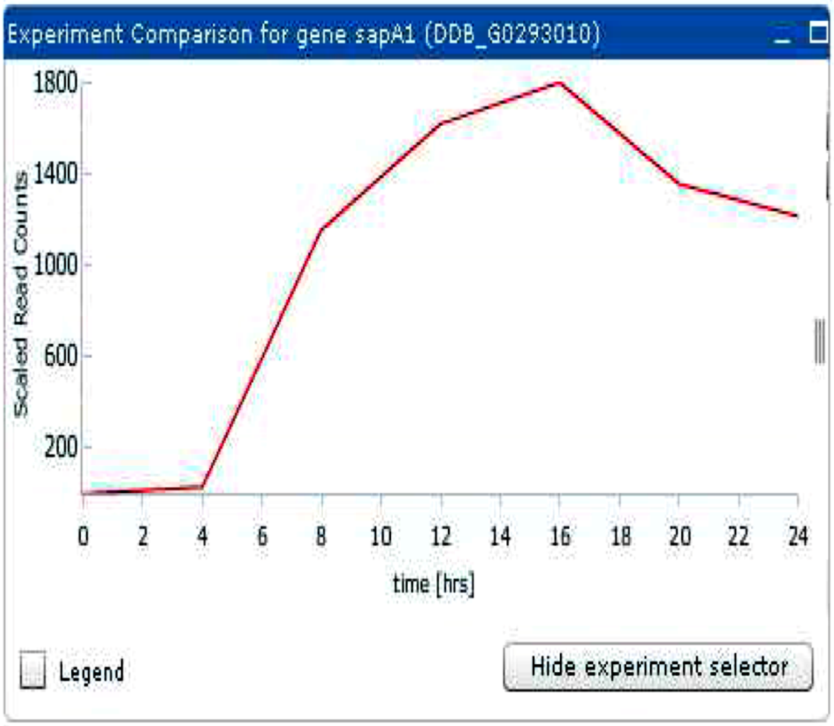

Supplementary Figure 6: Transcriptional regulation of *aplD* during *D. discoideum* development. Ax4 cells were grown on *K. aerogenes*, washed with KK2 buffer and allowed to develop on non-nutrient KK2 agar plates. Transcription profile of *aplD* was measured at time points indicated (Data adapted from Dicty express: https://dictyexpress.research.bcm.edu/bcm/#/all?genes=DDB_G0293010).).

Supplementary Table 1

Table 1: List of bacterial strains used in the study

**Bacterial strains**	**Phenotypes**
B. subtilis (*Bs*)	Non-pathogenic laboratory strain, ATCC 6051
E. coli B/r (*Ec* Br)	Non-pathogenic food bacteria for *D. discoideum* (Dicty stock centre)
K. aerogenes (*Ka*)	Non-pathogenic laboratory strain, ATCC 13883
K. pneumoniae (*Kp*52145)	Virulent, encapsulated bacteria with plasmid mediated virulence (Nassif et al., 1989) and (Benghezal et al., 2006).
K. pneumoniae (K^{-})	Mutant derivative of *Kp*52145, defective for capsule synthesis (Nassif et al., 1989).
K. pneumoniae (*Kp*LM21)	Clinical isolate which produces huge polysaccharide capsule, belongs to serotype K35, typed at the World Health Organisation International *Escherichia* and *Klebsiella* Centre, Copenhagen, Denmark [(Favre-Bonté et al., 1999) and (Balestrino et al., 2005)].
P. aeruginosa (PT531)	Quorum sensing mutant defective for *rhlR* and *lasR* genes (Cosson et al., 2002)
K. pneumoniae (*Kp*GFP)	*K. pneumoniae* expressing GFP reporter (Lee and Falkow, 1998; Benghezal et al., 2006)
E. coli (*Ec* DsRed)	*E. coli* expressing DsRed reporter; laboratory strain
B. megaterium	Non-pathogenic laboratory strain, ATCC 14581
